# Deciphering the Astrocyte Reaction in Alzheimer’s Disease

**DOI:** 10.3389/fnagi.2018.00114

**Published:** 2018-04-25

**Authors:** Beatriz G. Perez-Nievas, Alberto Serrano-Pozo

**Affiliations:** ^1^Institute of Psychiatry, King’s College London, London, United Kingdom; ^2^Alzheimer’s Research Unit, MassGeneral Institute for Neurodegenerative Diseases (MIND), Department of Neurology, Massachusetts General Hospital, Boston, MA, United States

**Keywords:** Alzheimer’s disease, amyloid plaques, astrocytes, glia, microglia, neurofibrillary tangles

## Abstract

Reactive astrocytes were identified as a component of *senile* amyloid plaques in the cortex of Alzheimer’s disease (AD) patients several decades ago. However, their role in AD pathophysiology has remained elusive ever since, in part owing to the extrapolation of the literature from primary astrocyte cultures and acute brain injury models to a chronic neurodegenerative scenario. Recent accumulating evidence supports the idea that reactive astrocytes in AD acquire neurotoxic properties, likely due to both a gain of toxic function and a loss of their neurotrophic effects. However, the diversity and complexity of this glial cell is only beginning to be unveiled, anticipating that astrocyte reaction might be heterogeneous as well. Herein we review the evidence from mouse models of AD and human neuropathological studies and attempt to decipher the main conundrums that astrocytes pose to our understanding of AD development and progression. We discuss the morphological features that characterize astrocyte reaction in the AD brain, the consequences of astrocyte reaction for both astrocyte biology and AD pathological hallmarks, and the molecular pathways that have been implicated in this reaction.

## Introduction

The term “glia” was first coined by Virchow to refer to the non-neuronal cells that form the “glue” of the brain (Virchow, 1858). Different types of glial cells were distinguished in the early years, including astroglia (van Lenhossék, 1895), microglia (del Río-Hortega and Penfield, 1892) and oligodendroglia (del Río-Hortega, 1921).

Astroglia or astrocytes were named after their stellate shape under the microscope. Soon after the development of appropriate staining methods, it became apparent that both acute (i.e., traumatic brain or spinal cord injury, stroke) and chronic (epilepsy, neurodegenerative diseases) insults to the central nervous system (CNS) are associated with a dramatic change in astrocyte morphology. In these conditions astrocytes appear hypertrophic and overexpress two intermediate-filament proteins of their cytoskeleton: glial fibrillar acidic protein (GFAP) and vimentin. These two characteristics qualify the astrocytes as “reactive”, as opposed to “resting” non-reactive astrocytes, which are not truly quiescent but exert many of the functions listed below. We prefer the terms astrocytic “reaction” or “response” over “astrocytosis” or “astrogliosis”, because the suffix *–osis* implies a pathological state of astrocytes or astroglia, which at present is not well characterized.

In this review article, we will first summarize the current knowledge about the physiological roles of astrocytes. A review of the morphological and molecular basis of astrocyte reaction in Alzheimer’s disease (AD) will follow next, with special emphasis in its consequences for AD pathophysiology. We will highlight the main ongoing scientific controversies and the remaining areas of uncertainty.

## Astrocytes in the Healthy Brain

### Morphological and Molecular Heterogeneity of Astrocytes

**Figure 1:**
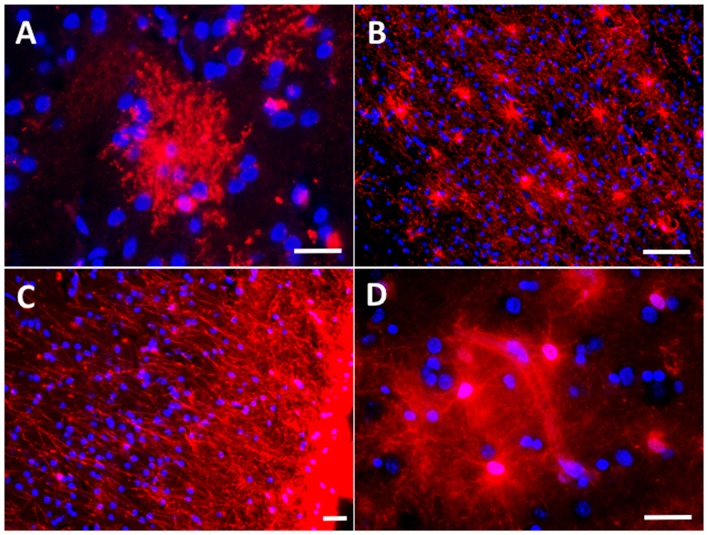
Astrocyte morphological features in the normal brain. **(A)** Protoplasmic astrocyte in layer II of the occipital neocortex with its typical bushy appearance depicted with immunohistochemistry for the glutamate transporter GLT-1/EAAT2. **(B)** Fibrous astrocytes in the white matter of the temporal lobe are rich in glial fibrillary acidic protein (GFAP). **(C)** Subpial interlaminar astrocytes in the frontal association neocortex of a healthy control individual form a palisade of processes which extend towards deep layers perpendicularly to the cortical surface and are GFAP-immunoreactive. **(D)** Perivascular astrocytes with their vascular endfeet wrapping a capillary vessel, here shown with immunohistochemistry for aldehyde dehydrogenase 1 L1 (constitutively present in all astrocytes). Scale bars: 10 μm in **(A,C,D)**; 20 μm in **(B)**.

Traditionally astrocytes have been classified in protoplasmic and fibrous. Protoplasmic astrocytes are cortical astrocytes, with no or minimal GFAP immunoreactivity in normal conditions, and with a “bushy” appearance owing to the profuse ramification of their processes in fine prolongations or leaflets, which reach the pre-and post-synaptic elements of the neurons (Figure 1A). By contrast, fibrous astrocytes are GFAP-immunoreactive astrocytes found in the white matter along the myelinated axons (Figure 1B). Research interest has focused by far on protoplasmic cortical astrocytes, in detriment of fibrous white matter astrocytes. Subpial interlaminar astrocytes are a third kind of astrocyte (Figure 1C). These are exclusively found in the most superficial layer of the cortex of primates and are characteristic for the flat shape of their soma and their long perpendicular processes towards the layers III and IV of the cortex (Colombo et al., 2000; Oberheim et al., 2009).

However, the morphological, functional and molecular heterogeneity of astrocytes is much broader and just starting to emerge (Sosunov et al., 2014; Chai et al., 2017; John Lin et al., 2017). For example, using the extracellular matrix receptor CD44 as pan-astrocytic marker, cortical and hippocampal human astrocytes have been recently classified in CD44+ astrocytes with and without long processes. CD44+ astrocytes with long processes are distinct from protoplasmic astrocytes in that they are located in the subpial layer (interlaminar astrocytes), deep cortical layers and hippocampus, and express high levels of GFAP and S100β and low levels of glutamine synthetase and glutamate transporters. CD44+ astrocytes without long processes are variable in shape and number and display a mixed phenotype between protoplasmic and fibrous astrocytes (Sosunov et al., 2014). A regional network-specific specialization of astrocytes has recently been unveiled. For example, mouse striatal and hippocampal astrocytes differ in some morphological aspects (higher astrocyte-neuron ratio in striatal, shorter astrocyte-synapse distance in hippocampal), electrophysiological properties (calcium spontaneous and evoked responses in hippocampal astrocytes), and transcriptomic and proteomic signature (Chai et al., 2017). Up to five distinct subpopulations of astrocytes have been distinguished in the mouse CNS, with variable proportions depending on the CNS region, each characterized by a characteristic transcriptomic program, and a different migration, proliferative and synaptogenic potential during development (John Lin et al., 2017).

### Physiological Roles of Astrocytes

A prolific research in the last two decades has expanded the role of astrocytes from a mere structural function to critical functions in CNS development, modulation of synaptic activity and glutamate homeostasis, blood-brain barrier (BBB) formation and neurovascular coupling, and inflammatory response. The basis of this astrocyte functional specialization remains largely unraveled, but it is plausible that specific subpopulations of astrocytes with distinct morphology and molecular equipment carry out different functions in different neural circuits (Chai et al., 2017).

#### Neurodevelopment

The refinement of the neural circuits during CNS development requires axon growth and synapse formation as well as pruning of redundant unnecessary synapses and axons. Recently, both microglia and astrocytes have been implicated in these processes. Astrocytes have been shown to promote the formation of excitatory synapses during CNS development (Allen et al., 2012) and to engulf and eliminate both excitatory and inhibitory synapses during prenatal development, and also in the adult brain (Chung et al., 2013).

#### Synaptic Function

Approximately 60% of excitatory synapses in the CA1 region of the rat hippocampus are tripartite, that is, have an astrocyte leaflet next to the presynaptic bouton and the postsynaptic dendritic spine (Ventura and Harris, 1999). Both cortical and hippocampal astrocytes are distributed in essentially non-overlapping domains (so called “islands”) with very little inter-digitation between their fine processes, a phenomenon termed “tiling” (Bushong et al., 2002; Halassa et al., 2007). In the mouse cortex, each astrocyte wraps an average of four neurons and up to 600 dendrites from different neurons (Halassa et al., 2007). The intimate relationship between astrocytes and neurons and the ratio astrocyte-neuron enable astrocytes to coordinate synaptic networks.

**Figure 2:**
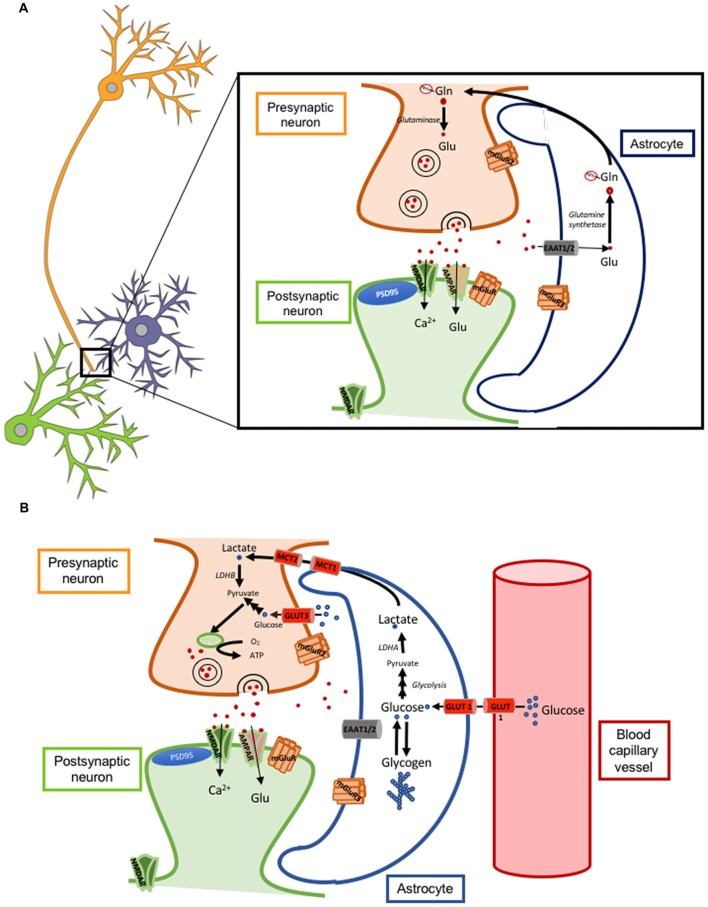
Astrocyte-neuron glutamate-glutamine cycle and lactate shuttle. **(A)** Cartoon illustrating a tripartite excitatory synapse with the glutamate-glutamine cycle. **(B)** Cartoon illustrating a tripartite excitatory synapse and a glioneurovascular unit with the lactate shuttle. Abbreviations: AMPAR, α-amino-3-hydroxy-5-methyl-4-isoxazolepropionic acid receptor; ATP, adenosine triphosphate; EAAT, excitatory amino acid transporter; Gln-NH3, glutamine; Glu, glutamate; GLUT, glucose transporter; LDH, lactate dehydrogenase; MCT, monocarboxylate transporter; mGluR, metabotropic glutamate receptor; NMDAR, N-methyl-D-aspartate receptor.

Astrocytes can modulate synaptic transmission and plasticity mainly by the re-uptake of glutamate from the synaptic cleft through their membrane glutamate transporters GLT-1 (also called excitatory amino acid transporter 2 or EAAT2; Figure 1A) and GLAST (also called excitatory amino acid transporter 1 or EAAT1). GLT-1 is more abundant than GLAST. Of note, the degree of astrocytic coverage of synapses is thought to be changing and this dynamic process can also impact the concentration and time course of glutamate at the synaptic cleft. If the coverage is reduced or there is a down-regulation of glutamate transporters, the resulting increased glutamate concentration for a prolonged time can cause either its spillover and activation of extra-synaptic neuronal NMDA receptors, leading to neuronal excitotoxicity, or its binding to presynaptic neuronal metabotropic glutamate receptors (mGluRs class III) leading to inhibition of glutamate release from the presynaptic neuron (Oliet et al., 2001; Figure 2A).

In addition, several groups have proposed that astrocytes can release small molecules that act as modulators of synaptic activity, a phenomenon they termed *gliotransmission*. The main purported gliotransmitters are adenosine triphosphate (ATP) and D-serine. During long term potentiation (LTP), astrocytes would release ATP to the synaptic cleft, which would be then rapidly hydrolyzed to adenosine by extracellular ectonucleotidases. Adenosine would bind A1 adenosine receptors in neurons to suppress excitatory transmission in neighboring non-stimulated pathways, a function named *heterosynaptic depression* (Pascual et al., 2005). On the other hand, astrocytes would synthesize and release D-serine, which would bind the glycine site in the NMDA receptor of post-synaptic neuron, a binding that is necessary for the opening of the NMDA receptor gate upon glutamate binding and for LTP (Henneberger et al., 2010). Extracellular D-serine is at least as abundant as glycine but an up to three times more potent ligand of the glycine site. To produce D-serine, astrocytes would uptake L-serine from the extracellular space and convert it with the cytosolic enzyme serine racemase using pyridoxal 5’-phosphate as cofactor, whereas D-serine degradation is carried out by the also cytosolic enzyme D-amino acid oxidase.

Recent studies, however, have challenged the concept of gliotransmission and a heated debate is currently ongoing (Fiacco and McCarthy, 2018; Savtchouk and Volterra, 2018). The gliotransmission body of evidence was largely built upon observations of *in vitro* studies using primary astrocyte cultures as well as *in vivo* studies using a dominant-negative SNARE (dnSNARE) mouse model under the GFAP promoter to suppress synaptic-like vesicle release specifically in astrocytes (Pascual et al., 2005). The observation that this promoter is leaky to neurons, that is, the dnSNARE transgene was largely expressed by neurons rather than astrocytes, has essentially invalidated many of the above reports (Fujita et al., 2014; Sloan and Barres, 2014). Similarly, the notion that D-serine is produced and released by astrocytes has recently been challenged (Wolosker et al., 2016). Using BAC-transgenic mice expressing enhanced green fluorescent protein (eGFP) under the serine racemase promoter (*Srr*), Ehmsen et al. (2013) elegantly showed that serine racemase is expressed almost exclusively in neurons. In any case, D-serine levels have been reported to be unchanged in soluble cortical extracts from AD subjects compared to aged controls (Chouinard et al., 1993; Nagata et al., 1995).

#### Neurovascular Unit

The astrocytic endfeet are a structural part of the BBB together with the endothelial cells, basement membrane and pericytes (Figure 1D). Together with pericytes (Hall et al., 2014), astrocytes are thought to help coordinate blood flow with neuronal activity, a concept known as *neurovascular coupling* (Mishra et al., 2016). In addition, astrocytes control water flux between the brain and the bloodstream through surface water channels called aquaporins (AQ), especially AQ1 (also expressed in choroid plexus) and AQ4 (also expressed in ependymal cells). Moreover, astrocytes have recently been involved in the paravascular clearance of toxic solutes through the expression of AQ4 in the astrocyte perivascular endfeet. The discovery of this mechanism was possible by tracking small fluorescent tracers injected in the subarachnoid space of living mice with multiphoton microscopy through a craniotomy. This novel drainage system, proposed by Nedeegaard and termed *glymphatic pathway*, posits that the arterial pulse wave within the brain determines the rapid movement of cerebrospinal fluid (CSF) from the subarachnoid cortical space into the paravascular space of the penetrating arteries, and from them to the capillary beds and the interstitial fluid (ISF), where an exchange of toxic solutes would take place. Efflux of these solutes would then occur though the paravenous spaces. Sleep, anesthesia, exercise, body posture—supine and specially lateral positions—facilitate this glymphatic transport, whereas sleep deprivation and prone position reduce its rate (Xie et al., 2013; Lee et al., 2015; He et al., 2017; von Holstein-Rathlou et al., 2018). This clearance system has been reported to largely rely on the expression of AQ4 in the astrocyte perivascular endfeet, because it is severely impaired in AQ4 knock-out mice (Iliff et al., 2012, 2013). The implications of these findings in AD pathophysiology will be discussed in detail below.

#### Energy Metabolism

Glucose is the main source of energy for the brain. Glucose utilization has been traditionally correlated with neuronal activity and, since the mid 1980s, the radiotracer 18-fluorodeoxyglucose ([^18^F]-FDG) has been used for PET imaging of neuronal activity and for the diagnosis of AD, where there is a typical symmetric bilateral temporo-parietal hypometabolism. However, a recent study with micro-PET in rats has elegantly demonstrated that astrocytes also actively uptake glucose and contribute, at least to some extent, to the brain [^18^F]-FDG PET signal (Zimmer et al., 2017). Of note, the uptake of glucose by astrocytes is coupled with the uptake of glutamate, because blocking the glutamate transporter GLT-1 prevents glucose utilization and aerobic glycolysis in astrocytes (Zimmer et al., 2017). Astrocytes store the glucose in the form of glycogen and contribute to fuel neurons by supplying lactate in certain situations such as hypoglycemia and ischemia, a phenomenon often referred to as *lactate shuttle* (Mächler et al., 2016; Figure 2B). Of note, lactate has been reported to be necessary for LTP and memory formation by supplying energy to the neurons (Suzuki et al., 2011).

## Morphological Features of Astrocyte Reaction in Alzheimer’s Disease

### Reactive Astrocytes Associate With Alzheimer’s Pathological Hallmarks

AD is the most common neurodegenerative disease and the most common cause of dementia. Pathologically, AD is defined by the presence of two core lesions: amyloid plaques and neurofibrillary tangles (NFTs). Amyloid plaques are extracellular deposits of the *amyloid β peptide* (Aβ), which is a normal by-product resulting from the sequential cleavage of the transmembrane protein amyloid β precursor protein (AβPP) by the aspartyl-proteases β- and γ-secretases. NFTs are intracellular inclusions of the microtubule associated protein *tau*, which in the AD brain is aberrantly hyperphosphorylated and misfolded. Besides the development of these lesions, AD is associated with the disappearance of synapses, dendritic branches and neurons (Serrano-Pozo et al., 2011a). The first descriptions of the existence of prominent cortical astrocytic and microglial reactions in the AD brain date from the late 1980s (Beach and McGeer, 1988; Beach et al., 1989; Itagaki et al., 1989), but amyloid plaques, NFTs, and their effects on neurons and synapses have monopolized researchers’ interest for a long time, and the role of non-neuronal cells such as microglia and astrocytes is only recently gaining scientific momentum.

**Figure 3:**
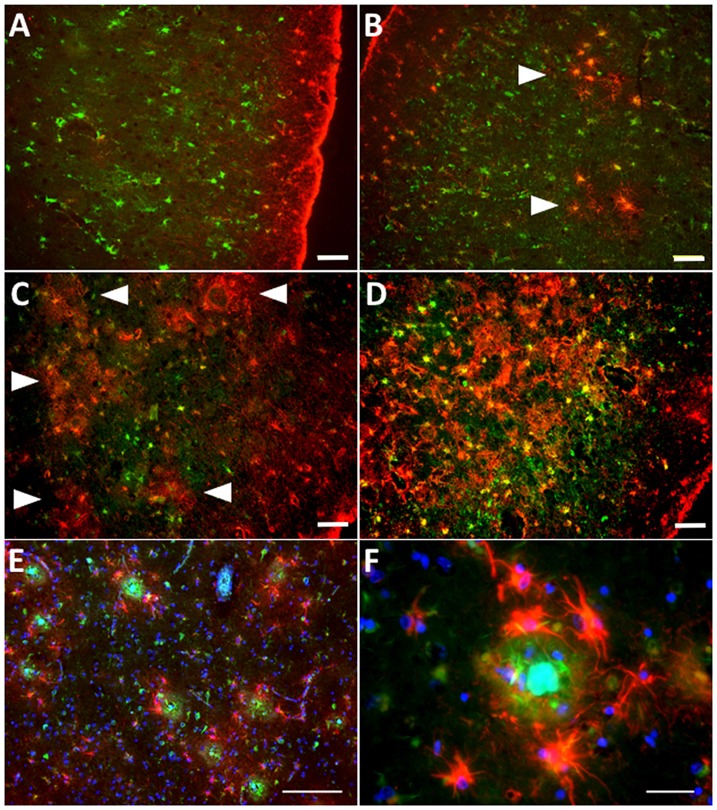
Astrocyte reaction to amyloid plaques in the Alzheimer’s brain. **(A–D)** Double fluorescent immunohistochemistry for GFAP (red) and the enzyme glutamine synthetase (green) showing the progression of astrocyte reaction (defined by GFAP immunoreactivity) in the temporal association neocortex (Brodmann’s area 38) of a healthy control subject without Alzheimer’s disease (AD) changes **(A)**, another healthy control individual with sparse neuritic plaques **(B)**, and two patients with an AD diagnosis **(C,D)**. Arrowheads point to increasingly numerous clusters of GFAP-immunoreactive astrocytes, which become confluent in advanced stages of the disease. **(E)** Clusters of GFAP-immunoreactive astrocytes (red) surrounding dense-core amyloid plaques (Thioflavine-S positive, green) in the temporal association neocortex (Brodmann’s area 38) of an AD patient. **(F)** GFAP-immunoreactive astrocytes penetrate and surround dense-core amyloid plaques with their processes. Scale bars: 50 μm in **(A–D)**, 100 μm in **(E)**, and 10 μm in **(F)**.

While reactive astrocytes are not exclusive of AD, the relationship between reactive astrocytes and AD pathological hallmarks, particularly amyloid plaques, is the most intimate in any neurodegenerative disease. In fact, senile amyloid plaques are defined among other features by the presence of a cluster of reactive astrocytes that penetrate and embrace amyloid deposits with their processes, fragmenting and isolating plaques from the surrounding neuropil (Itagaki et al., 1989; Figures 3A,B), and reactive astrocytes follow the laminar distribution of amyloid plaques in the association cortex (Beach and McGeer, 1988). Postmortem quantitative neuropathological studies have shown that the number of reactive astrocytes in the vicinity of amyloid plaques increases as the disease advances (Pike et al., 1995; Vehmas et al., 2003) and is independent of plaque size and apolipoprotein E (*APOE*) genotype (Figures 3C–F).

**Figure 4:**
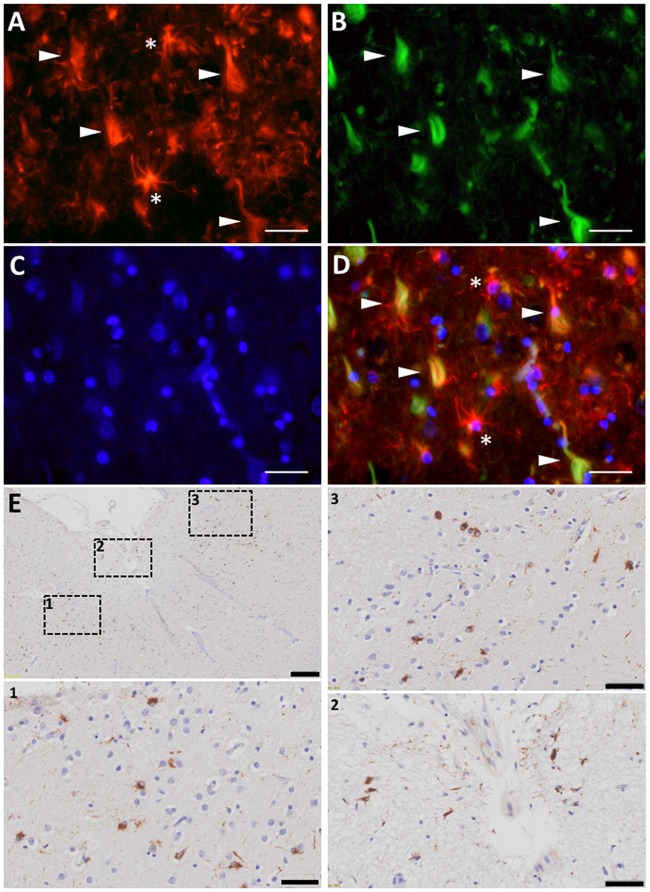
Astrocyte reaction to neurofibrillary tangles (NFTs) in the Alzheimer’s brain. **(A–D)** Fluorescent immunohistochemical staining for GFAP (**A**, red) with Thioflavine-S (**B**, green) and DAPI (**C**, blue) staining in the temporal association neocortex (Brodmann’s Area 38) of an individual with a diagnosis of AD. Reactive (GFAP+) astrocytes surround and penetrate extracellular “ghost” NFTs, so that these late-stage extracellular NFTs become GFAP-immunoreactive (**D**, merge). **(E)** Peroxidase-DAB immunohistochemical staining for total tau showing thorn-shaped tau-immunoreactive astrocytes in the subpial (inset 2) and mid layers (insets 1 and 3) of the frontal association cortex (Brodmann’s areas 8/9) of a subject with a diagnosis with AD. Scale bars: 10 μm in **(A–D)**, 200 μm in **(E)**, and 50 μm in insets 1–3.

The association between reactive astrocytes and NFTs—the other core pathological lesion of the disease—has received much less attention, not only in AD but also in all the other tauopathies such as Pick’s disease, progressive supranuclear palsy (PSP), corticobasal degeneration (CBD), argyrophilic grain disease (AGD), or chronic traumatic encephalopathy (CTE). However, immunohistochemical and electron microscopy studies have shown that reactive astrocytes can also penetrate with their processes the extracellular “ghost” NFTs present in the midst of the neuropil in advanced AD (Ikeda et al., 1992a,b; Figures 4A–D). Therefore, these end-stage NFTs can exhibit both tau and GFAP immunoreactivities (Probst et al., 1982; Irwin et al., 2012). Postmortem quantitative neuropathological studies have shown that this spatial association between reactive astrocytes and NFTs also parallels the progression of the disease (Simpson et al., 2010).

### Migration or Just Reorientation of Processes?

When astrocytes respond to an experimental acute injury and become reactive, they occupy the same neuropil volume and do not lose their even and non-overlapping distribution within the neuropil despite the hypertrophy and increased number of their main processes (Wilhelmsson et al., 2006). Whether reactive astrocytes migrate towards amyloid plaques or just reorient their processes towards them has not been investigated in detail. This distinction is important because the physiologic distribution of astrocytes in non-overlapping domains described above is thought to be crucial for their homeostatic and synaptic functions.

Several studies provide indirect evidence that astrocytes direct their processes but do not substantially move their cell bodies towards the plaques. The observation that the number of reactive astrocytes does not correlate with plaque size argues against their chemotactic migration towards plaques and in favor of a reaction of local “resting” or homeostatic astrocytes. A more recent statistical physics-based spatial analysis of astrocytes imaged *in vivo* in the cortex of APPswe/PS1dE9 and wild-type littermates confirmed that plaques in these transgenic mice do not alter the spatial distribution of astrocytes much (Galea et al., 2015). By contrast, homeostatic microglial cells are known to be highly dynamic and motile surveillants of the brain (Nimmerjahn et al., 2005), activated microglial cells have been shown to migrate towards dense-core amyloid plaques in mice* in vivo* (Meyer-Luehmann et al., 2008), and the number of activated microglial cells in the vicinity of plaques does correlate with plaque size, supporting a chemotactic effect (Serrano-Pozo et al., 2013b).

The reorientation of astrocyte processes towards amyloid plaques is dependent on the intermediate filaments of the cytoskeleton because depletion of GFAP and vimentin from astrocytes in a mouse model of AD causes a failure of astrocytes to penetrate within plaques and a decrease in astrocyte-plaque overlap (Kraft et al., 2013; Kamphuis et al., 2015). This phenomenon is so prominent that the palisade of long interlaminar astroglial processes, characteristic of the supragranular layers of the isocortex of primates, is severely disrupted or even virtually absent in AD and Down’s syndrome patients with AD neuropathological changes (Colombo et al., 2000, 2002, 2005). Importantly, these interlaminar astrocytes are likely major contributors to the microcolumnar functional organization of neurons in the isocortex (perpendicular to the pial surface), which is disrupted in AD, with thinner microcolumns and wider spaces between them (Buldyrev et al., 2000).

The up-regulation of GFAP in reactive astrocytes can be so dramatic in advanced AD that the GFAP protein can deposit in the astrocyte primary processes forming eosinophilic elongated structures called Rosenthal fibers (Wegiel and Wisniewski, 1994). These Rosenthal fibers are very much like those that define Alexander’s disease, a rare, usually childhood-onset, progressive leukodystrophy that causes spastic quadriparesis, seizures and intellectual decline. Interestingly, Alexander’s disease is due to mutations in the *GFAP* gene that result in decreased solubility and subsequent deposition of the GFAP protein within the cytoplasm of the white matter fibrous astrocytes (Hsiao et al., 2005; Hagemann et al., 2006). The insolubility of GFAP in advanced AD and Alexander’s disease could have deleterious effects in astrocyte biology by both overwhelming their protein degradation systems, autophagy (Tang et al., 2008) and proteasome (Tang et al., 2010; Orre et al., 2013), and restricting their range of migration and process motion.

### Proliferation or Just Phenotypic Change?

Because reactive astrocytes are conventionally identified by their GFAP immunoreactivity and GFAP expression in non-reactive (resting) astrocytes is often below the detection level of immunohistochemistry, it is not uncommon to misinterpret the enhanced GFAP immunoreactivity seen in the AD brain as evidence of astrocyte proliferation. A few studies have found increased expression of cell division markers (i.e., cyclins, PCNA, Ki67) within astrocytes in the brain of AD subjects (Nagy et al., 1997; Wharton et al., 2005).

Despite these studies, there is more substantial and stronger evidence against proliferation of astrocytes or glial progenitors in AD. Experiments injecting BrdU in plaque-bearing mice have definitively demonstrated that only microglia exhibits significant proliferation, particularly in the proximity of plaques (Bondolfi et al., 2002; Kamphuis et al., 2012; Luccarini et al., 2012; Sirko et al., 2013). The same conclusion was achieved by a recent human postmortem study using the proliferative marker PCNA, Aβ and Iba1 or GFAP triple immunohistochemistry (Marlatt et al., 2014). Similar numbers of GFAP-positive astrocytes were found in wild-type and 3xTg AD mice hippocampus, where >80% astrocytes are GFAP-immunoreactive (Olabarria et al., 2010). Last, the number of cortical astrocytes was found to be similar between healthy and AD brains in two stereology-based quantitative human postmortem studies: one using morphological identification of astrocytes in Nissl-stained sections (Pelvig et al., 2003), and another using double fluorescent immunohistochemistry with GFAP and aldehyde dehydrogenase 1 isoform L1 (ALDH1L1) or glutamine synthetase as pan-astrocytic constitutive markers (Serrano-Pozo et al., 2013a).

### Immortal or Senescent and Mortal?

Cell death is the other side of the coin with respect to cell proliferation. The finding of a similar number of astrocytes in AD and normal brains and the lack of correlation between total astrocyte number and AD progression could have two interpretations: (1) there is no significant astrocyte proliferation or death in either the normal or the AD brain; and (2) there is significant proliferation and death but both rates are similar. Several postmortem studies using terminal transferase-mediated dUTP-biotin nick end labeling (TUNEL) and caspase 3 activated immunohistochemistry (Smale et al., 1995; Li et al., 1997; Kobayashi et al., 2004) have reported apoptotic astrocytes, but other authors have not found astrocyte apoptosis (Sugaya et al., 1997). These studies are likely confounded by the cause of death and the potential pro-apoptotic effects of the agonal period immediately before death. The finding of increased CSF levels of astrocytic markers in AD patients with respect to healthy control subjects, including GFAP (Jesse et al., 2009; Ishiki et al., 2016) and glutamine synthetase (Gunnersen and Haley, 1992; Tumani et al., 1999), argues in favor of some astrocyte cell death.

Another possibility is that astrocytes reach a senescent state in the AD brain. Morphologically, senescent-looking astrocytes have been characterized in the hippocampus and entorhinal cortex of 3xTg AD mice as astrocytes that are located far from amyloid plaques (>50 μm), with atrophied cell somas, and simplified processes (Olabarria et al., 2010; Yeh et al., 2011). However, other authors have reported that the size of astrocyte somas far from amyloid plaques (>60 μm) in an APP/PS1 transgenic mouse model is comparable to that of wild-type mice (Brawek et al., 2018). Another more dramatic morphological change that may resemble astrocyte senescence is the so-called *clasmatodendrosis*. Clasmatodendritic astrocytes are characteristic for perikaryal swelling and accumulation of lysosomes and vacuoles, condensed chromatin leading to pyknotic nuclei, and fragmented or beaded processes. Recently, astrocytes expressing green-fluorescent protein (GFP) under the GFAP promoter have been reported to be sensitive to clasmatodendrosis in a plaque-bearing AD mouse model (Daschil and Humpel, 2016). However, it should be noted that this phenomenon has been described mostly in the fibrous astrocytes from periventricular and deep white matter lesions of individuals with vascular dementia and/or AD, rather than in cortical astrocytes. Moreover, the development of clasmatodendrosis has been attributed to ischemia, hypoxia, local disruption of the local BBB and acidification of the local environment, rather than to the toxicity of AD pathological hallmarks (Tomimoto et al., 1997; Sahlas et al., 2002; Chen et al., 2016). p16^INK4a^ and matrix metalloproteinase 1 (MMP1) have been proposed as molecular markers defining senescent astrocytes (Bhat et al., 2012).

## Functional Consequences of Astrocyte Reaction in Alzheimer’s Disease

As with the morphological aspects, considerable controversy surrounds the topic of the functional consequences of astrocyte reaction in AD. Conceptually, AD-associated astrocyte reaction could entail a loss of the normal functions of the astrocyte (i.e., modulation of synaptic function, BBB integrity and function), a gain of a new toxic function (i.e., inflammation), or both.

### Plaque Formation or Plaque Clearance?

Whether reactive astrocytes contribute to plaque formation and maturation or to Aβ clearance and plaque growth restriction is still debated. Multiple immunohistochemical and immunoelectron microscopy postmortem studies on the AD brain have revealed that astrocytes and, to a lesser extent, microglia, contain granules of non-fibrillar Aβ, presumably engulfed from extracellular diffuse amyloid deposits (Akiyama et al., 1996, 1999; Funato et al., 1998; Yamaguchi et al., 1998; Thal et al., 2000; Oide et al., 2006). However, this observation has been also interpreted as evidence for an active participation of reactive astrocytes in plaque formation (Nagele et al., 2003), rather than clearance. A feed-forward mechanism has been proposed by which reactive astrocytes could up-regulate BACE1 and promote the amyloidogenic processing of AβPP in response to noxious stimuli such as inflammation (Zhao et al., 2011) or ischemia (Hartlage-Rübsamen et al., 2003). It is noteworthy that Aβ-containing astrocytes have been found preferentially near fleecy N-terminally truncated Aβ deposits in the human entorhinal cortex (Thal et al., 2000) and that astrocytes have been implicated in the generation and secretion of N-terminally truncated Aβ in a BACE1-independent manner (Oberstein et al., 2015).

On the other hand, *ex vivo* and *in vivo* experiments applying adult mouse or human astrocytes on the brain of plaque-bearing mice have confirmed that astrocytes uptake and clear Aβ, with diffuse deposits over larger fibrillar aggregates being preferentially removed (Koistinaho et al., 2004; Pihlaja et al., 2008, 2011; Nielsen et al., 2009, 2010), These experimental studies are in agreement with a postmortem immunohistochemical study that has confirmed the presence of oligomeric protofibrillar forms of Aβ within reactive astrocytes using conformation-specific antibodies (Lasagna-Reeves and Kayed, 2011). Two main mechanisms have been proposed: secretion of Aβ-degrading enzymes and phagocytosis (uptake and lysosomal degradation).

The phagocytosis of Aβ by astrocytes requires the participation of APOE because APOE null astrocytes are not efficient at amyloid plaque removal (Koistinaho et al., 2004). Using intracortical microinjection of fluorescently-labeled Aβ and *in vivo* multiphoton microscopy through a craniotomy in 5xFAD mice—characterized by a fast amyloid plaque deposition, it has been shown that the phagocytic potential of astrocytes declines with aging (Iram et al., 2016). Interestingly, in these mice the complement factor C1q can facilitate Aβ phagocytosis by astrocytes (Iram et al., 2016). Enhancing lysosomal biogenesis in astrocytes through viral delivery of the transcription factor EB (TFEB) results in increased Aβ phagocytosis and degradation in APP/PS1 mice (Xiao et al., 2014).

Postmortem immunohistochemical studies have revealed that astrocytes express some of the known Aβ-degrading enzymes. For example, endothelin-converting enzyme-2 (ECE-2) is up-regulated in AD astrocytes, although also in neurons and some microglial cells (Palmer et al., 2009). MMP3 is present in plaques and in white matter fibrous astrocytes in the AD brain (Yoshiyama et al., 2000; but see also, Baig et al., 2008). Astrocyte deficiency of lipoprotein-related protein 1 (LRP1), an apoE receptor, reduces the secretion of Aβ-degrading enzymes such as matrix metalloproteases (MMPs) 6 and 9 and insulin degrading enzyme (IDE) and accelerates amyloid plaque deposition (Liu et al., 2017). Of note, IDE expression has been shown to increase in reactive astrocytes around plaques in the Tg2576 AD mouse model (Leal et al., 2006).

It should be noted that both amyloid plaque burden and plaque size distribution remain relatively stable throughout the disease progression, indicating that there is no significant net plaque growth and clearance, that is, the clearance mechanisms might just enable to neutralize growth but not to effectively eliminate plaques (Hyman et al., 1995; Serrano-Pozo et al., 2011b, 2012). Although depletion of GFAP and vimentin from astrocytes led to a failure of penetration into amyloid plaques in a mouse model of AD, it is uncertain whether plaque growth and amyloid deposition subsequently accelerate because the two existing studies showed conflicting results (Kraft et al., 2013; Kamphuis et al., 2015). Therefore, although there is no proportionality between plaque size and number of surrounding reactive astrocytes (Serrano-Pozo et al., 2013b), it is plausible that reactive astrocytes form an effective physical barrier around the plaques and limit their growth.

Interestingly, besides Aβ, astrocytes can accumulate other proteins associated with neurodegeneration such as tau and α-synuclein. The presence of tau-immunoreactive “tufted” astrocytes pathologically defines PSP, whereas the finding of tau-immunoreactive “astrocytic plaques” defines the pathological diagnosis of CBD. An aging-related tau astrogliopathy (ARTAG) has recently been described with “thorn-shaped” and “granular fuzzy” tau-positive astrocytes in the medial temporal lobe and other brain regions (Kovacs et al., 2016; Liu et al., 2016). These thorn-shaped astrocytes have also been identified in the brain of individuals with AD (Lace et al., 2012; López-González et al., 2013; Figure 4E). In fact, classic electron microscopy studies in the 1990s already described the presence of paired helical filaments of tau in astrocytes in AD (Ikeda et al., 1992a; Yamazaki et al., 1995; Arima et al., 1998). Of note, although NFTs in AD are composed of similar amounts of three repeat (3R) and four repeat (4R)-tau, astrocyte tau immunoreactivity in AD, ARTAG, and the classic tauopathies PSP and CBD is predominantly 4R-tau, not 3R. Whether astrocytes have a predilection for up-taking 4R-tau over 3R-tau, or whether they degrade 3R-tau more readily than 4R-tau, remains unknown. Similarly to tau, α-synuclein accumulation has been found within astrocytes in the basal forebrain of subjects with sporadic Parkinson’s disease (PD; Braak et al., 2007). Although it is possible that astrocytes express low levels of these genes and up-regulate their expression in these neurodegenerative diseases, it is more plausible that they take up misfolded forms of these proteins from the ISF, since both tau and α-synuclein are thought to be released by neurons at the synapse and to propagate from neuron to neuron trans-synaptically (Luk et al., 2012; de Calignon et al., 2012). Whether and how astrocyte reaction impacts the prion-like propagation of these proteins, and the consequences of these astrocyte proteinopathies for the astrocyte biology remain to be investigated.

### Neuroprotection or Neurotoxicity?

A simplistic view of reactive astrocytes as neuroprotective in AD alludes to the formation of a scar-like physical barrier between the amyloid plaques and the surrounding neuropil. Reactive astrocytes around amyloid plaques may, thus, limit the collateral damage from diffusible soluble Aβ oligomeric species by isolating the reservoirs of these especially neurotoxic Aβ species. For example, in a quantitative neuropathological study from the population-based Medical Research Council-Cognitive Function and Aging Study (MRC-CFAS), the number of diffuse and compact plaques lacking astrocyte reaction in layer VI of the cingulate cortex was independently associated with worse cognition (lower MMSE score; Mathur et al., 2015). The attenuation of astrocyte reaction by depletion of GFAP and vimentin led to a multiplication of plaque-associated dystrophic neurites, also suggesting a net neuroprotective effect of reactive astrocytes in AD (Kraft et al., 2013). On the other hand, reactive astrocytes around neuritic plaques have recently been shown to engulf plaque-associated dystrophic neurites of APPPS1 mice and AD patients and this phagocytic function has been deemed neuroprotective (Gomez-Arboledas et al., 2018). These observations contrast with other studies supporting the idea that astrocyte reaction contributes to neurodegeneration in dementia due to AD. Astrocytes isolated from aged 5xFAD offer much less neurotrophic support when co-cultured with neonatal neurons as compared with astrocytes from wildtype mice (Iram et al., 2016). Postmortem studies comparing subjects with dementia due to AD and cognitively intact subjects with high levels of amyloid plaques and NFTs (so called “high pathology control”, “asymptomatic AD”, or “mismatch AD” cases) have shown that the latter group lacks the prominent microglial and astrocyte responses typical of the AD brain, and have lower or normal levels of inflammatory cytokines and preserved neuron number and synaptic density (Lue et al., 1996; Perez-Nievas et al., 2013).

An unbiased way to approach this question is to study the transcriptomic profile of astrocytes from the AD brain compared to the normal aging brain. It should be noted that a substantial region-dependent shift in the astrocyte transcriptome has been reported with aging in both mouse and human normal brains (Soreq et al., 2017; Boisvert et al., 2018). Specifically, aged murine astrocytes down-regulate cholesterol synthesis and up-regulate synaptic elimination and immune pathways, whereas homeostatic and glutamate neurotransmission genes do not appear to change much with aging (Boisvert et al., 2018). Several human postmortem studies using laser capture microdissection (LCM) of astrocytes in brains from AD patients and non-demented healthy controls have investigated the transcriptomic changes that occur in AD astrocytes. Simpson et al. (2011) used GFAP to identify and capture astrocytes by LCM and compared the transcriptomic profile between subjects with an advanced Braak stage of NFTs (V–VI) and subjects with low Braak stages (I–II). They observed a dysregulation of genes associated with the actin cytoskeleton, proliferation, apoptosis, and ubiquitin-mediated proteolysis at low Braak stages, that contrasted with an altered regulation of intracellular signaling pathways, including insulin, phosphatidylinositol 3-kinase (PI3K)/Akt, and mitogen-activated protein kinase (MAPK) pathways at high levels of AD pathology. In another more recent LCM-based transcriptomic study using ALDH1L1 rather than GFAP as astrocytic marker, the authors found an up-regulation of genes encoding both astrocytic immune response and mitochondrial machinery in the posterior cingulate gyrus—an area of abundant and early Aβ deposition—of AD patients compared to healthy subjects (Sekar et al., 2015). The advantage of using ALDH1L1 instead of GFAP is that non-reactive “resting” GFAP-negative astrocytes are better represented in the healthy control group, allowing for the detection of more subtle differences in gene expression between health and disease.

Although meritorious, the results of human postmortem transcriptomic studies should be taken with caution because they can be affected by several confounders including the effects on gene expression of the cause of death and the agonal period prior to death (i.e., hypoxia, ischemia, sepsis), the postmortem interval, and the common presence of mixed pathologies such as Lewy bodies and cerebrovascular disease. Moreover, it is not easy to dissect the effect of amyloid plaques and NFTs on astrocyte gene expression. By contrast, transcriptomic studies in transgenic mice are devoid of these confounders. Orre et al. (2014) performed a transcriptional analysis on acutely isolated astrocytes from the cortex of aged controls and APPswe/PS1dE9 AD mice using GLT-1 as astrocytic marker for fluorescently-assisted cell sorting (FACS). These mice develop amyloid plaques similar to human AD plaques, but not NFTs. In this transgenic AD mouse model, astrocytes exhibited a proinflammatory immune phenotype and a reduced expression of neuronal support genes and genes involved in neuronal communication. Based on the distinct transcriptomic profiles observed in mouse models of acute brain injury, such as the lipopolysaccharide (LPS) model of neuroinflammation and the stroke model of middle cerebral artery occlusion, Barres proposed a classification of astrocytes in neurotoxic (A1) and neuroprotective (A2; Zamanian et al., 2012). These authors recently showed that, *in vitro*, A1 astrocytes lose their ability to promote neuronal survival, neurite outgrowth, synapse formation and phagocytosis, leading to neuron and oligodendrocyte death, and postulated that A1 astrocytes can be identified by the expression of complement fraction 3 (C3) and are abundant in many human neurological diseases such as AD, amyotrophic lateral sclerosis, Huntington’s disease, and multiple sclerosis (Liddelow et al., 2017). Following this classification, an age-dependent neurotoxic transcriptomic signature (A1) has recently been reported in astrocytes isolated from a tauopathy mouse model under the human *APOE*ε4 knock-in (KI) background, as compared with the *APOE*ε3 KI background (Shi et al., 2017).

Thus, taken together, astrocyte transcriptomic studies from human AD brain and AD mouse models support the idea that astrocyte reaction in AD involves a gain of neurotoxic function and loss of neuroprotective function of astrocytes.

### Effects on Synaptic Function

There is growing evidence indicating that astrocyte reaction impairs the normal function of astrocytes as modulators of neuronal synaptic transmission. Most studies have focused on glutamatergic transmission and have attributed reactive astrocytes a crucial participation in glutamate-mediated neuronal excitotoxicity.

Using* in vivo* multiphoton microscopy through a craniotomy and the glutamate-sensitive probe iGluSnFR delivered with an intracortical injection of an adeno-associated viral vector, Hefendehl et al. (2016) have recently reported that the microenvironment surrounding plaques in an APPPS1 mouse model has chronically elevated glutamate concentrations, and that neurons in the immediate vicinity of plaques do not appropriately respond to stimuli, such as hindlimb pinch in the somatosensory cortex or visual stimuli in the visual cortex, as judged by the *in vivo* imaging analysis of calcium transients and glutamate concentration. This increased peri-plaque glutamate concentration correlated with a reduced expression of GLT-1/EAAT2 in the reactive (GFAP+) astrocytes surrounding plaques, and was partially corrected by the intravenous administration of the antibiotic ceftriaxone, which is known to up-regulate the expression of GLT1-/EAAT2 by astrocytes (Hefendehl et al., 2016). Of note, soluble Aβ oligomers reduce the expression of the glutamate transporter GLT-1/EAAT2 in astrocyte cultures through a mechanism involving calcineurin (CN)/nuclear factor of activated T cells (NFAT) pathway (Abdul et al., 2009) and oxidative stress (Scimemi et al., 2013). This reduction of GLT-1/EAAT2 expression by astrocytes parallels the progression of AD pathology in the human brain (Simpson et al., 2010). Recently, it has been proposed that preservation of GLT-1/EAAT2 expression in GFAP+ reactive astrocytes could be a mechanism of resilience against AD neuropathological changes (Kobayashi et al., 2018). Other authors have shown a decreased solubility of GLT-1/EAAT2 in the brain of patients with AD, which could potentially impair the re-uptake of synaptic glutamate (Woltjer et al., 2010).

A second proposed mechanism for astrocyte-mediated neurotoxicity is the downregulation of glutamine synthetase expression level or activity, leading to a subsequent reduction in the astrocyte capacity to detoxify neuronal glutamate to glutamine. Indeed, acute viral-induced astrocyte reaction can induce neuronal glutamate excitotoxicity via down-regulation of the astrocyte cytosolic enzyme glutamine synthetase (Ortinski et al., 2010) and glutamine synthetase levels have been reported to be reduced in the 3xTg mouse prefrontal cortex (Olabarria et al., 2011; Kulijewicz-Nawrot et al., 2013). However, whether this is also the case in the human AD brain remains unclear (Le Prince et al., 1995; Tumani et al., 1999; Serrano-Pozo et al., 2013a). Notwithstanding these conflicting reports, glutamine synthetase catalytic activity has been shown to be sensitive to oxidation and may be impaired by oxidative damage in AD (Smith et al., 1991; Hensley et al., 1994, 1995). Perhaps as compensatory mechanism against glutamate excitotoxicity, glutamine synthetase can be up-regulated in groups of cortical pyramidal neurons in AD brains, although not selectively in the vicinity of plaques (Robinson, 2000; Serrano-Pozo et al., 2013a). The common denominator of all these mechanisms is thought to be an increase in extracellular glutamate levels leading to excessive activation of extra-synaptic NMDA receptors and, thereby, neuronal excitotoxicity (Li et al., 2011). This common mechanism could explain the finding of calcium overload (Kuchibhotla et al., 2008) and hyperactive neurons (Busche et al., 2008, 2012) found in the proximity of plaques using multiphoton calcium imaging in live APP/PS1 mice, although the latter finding was attributed to a decreased gabaergic inhibition instead of an excess of glutamate.

A third alternative novel mechanism proposed for astrocyte-mediated neurotoxicity is an enhancement of gabaergic tone, rather than excessive glutamatergic transmission or reduced gabaergic inhibition. Reactive astrocytes from plaque-bearing AD mouse models have been shown to induce neuronal tonic inhibition via increase in GABA release to the synaptic cleft (Mitew et al., 2013; Jo et al., 2014; Wu et al., 2014). A recent detailed histological study has described a transient increase in GABA immunoreactivity of astrocytes in middle aged (but not young or very old) APP/PS1 transgenic mice, specifically in reactive astrocytes surrounding dense-core plaques (Brawek et al., 2018). This mechanism could explain the co-existence of an abnormally high proportion of silent neurons in multiphoton calcium imaging studies on living APP/PS1 mice (Busche et al., 2008, 2012).

### Effects on Blood Brain Barrier Integrity and Function

Besides forming amyloid plaques in the brain parenchyma, Aβ can deposit in the wall of small cortical and leptomeningeal vessels and capillaries, a condition called cerebral amyloid angiopathy (CAA). CAA can occur without concomitant AD pathology, but more commonly is present in up to 90% of individuals with a postmortem diagnosis of AD, usually with a mild degree (Serrano-Pozo et al., 2011a). In addition to the possibility of causing lobar intracerebral and focal subarachnoid hemorrhages due to vessel rupture, CAA can cause brain hypoperfusion and subsequent ischemia, and independently contributes to AD-related cognitive decline (Neuropathology Group of the Medical Research Council Cognitive Function and Ageing Study, 2001; Pfeifer et al., 2002; Arvanitakis et al., 2011; Serrano-Pozo et al., 2013c). Moreover, an increased CSF/plasma albumin ratio, indicating increased BBB permeability, has been repeatedly shown in AD and other dementias (Skoog et al., 1998; Bowman et al., 2007; Skillbäck et al., 2017).

Since astrocytes are part of the BBB, the contribution of astrocyte reaction to BBB disruption and amyloid plaque and CAA accumulation are gaining increasing research interest. Does astrocyte reaction affect BBB integrity in AD and CAA? Indeed, a number of astrocyte abnormalities have been described in the neurovascular unit of mouse models of AD and CAA and human AD brains including: (1) swelling and detachment of astrocyte endfeet; (2) altered secretion of proteins that are part of the basement membrane or the extracellular matrix; (3) reduction of both endothelial and astrocytic glucose transporter 1 (Glut1); (4) reduced astrocytic expression of monocarboxylate transporter 1 (MCT1) resulting in a decreased lactate release; and (5) loss of potassium and water channels (AQ; Wilcock et al., 2009; Hawkes et al., 2011; Merlini et al., 2011). Furthermore, astrocyte-dependent cerebral vasoreactivity was reported to be impaired in amyloid-laden vessels in a mouse model of CAA (Kimbrough et al., 2015).

In addition, it is possible that astrocyte reaction impairs the clearance of Aβ at the BBB. Weller et al. (2008) proposed that Aβ is cleared from the ISF through a perivascular drainage from capillaries to cortical and leptomeningeal arteries through the basement membranes. They proposed that the suction effect after the arterial pulse wave drives this perivascular drainage and that the stiffening of the arterial walls with aging and vascular risk factors could attenuate this arterial pulsatility and favor Aβ accumulation in capillaries and arteries in the form of CAA and in the brain parenchyma in the form of amyloid plaques (Weller et al., 2008; Hawkes et al., 2011, 2014; Arbel-Ornath et al., 2013). In 2012, Nedergaard redefined this hypothesis by proposing that toxic solutes including Aβ are cleared from the ISF through a paravascular drainage system that involves astrocytes and would serve as the brain lymphatic system, hence the term *glymphatic* (Iliff et al., 2012, 2013). Specifically, the glymphatic system hypothesis postulates that there is an inflow of CSF from the subarachnoid space to the paravascular space of penetrating arteries, between the basement membrane of the endothelium and the smooth muscle cell layer; then, low molecular weight solutes including Aβ pass to the ISF through the astrocyte end-feet of the BBB and efflux from the brain parenchyma through the paravenous space by a convective bulk-flow driven by the arterial pulse wave and the arterio-venous pressure gradient, rather than simple diffusion. Noteworthy, sleep has been shown to potentiate this transport system (Xie et al., 2013), and sleep deprivation has been shown to enhance Aβ deposition (Kang et al., 2009). This glymphatic drainage system would be largely dependent on the expression of AQ4 in the astrocyte perivascular endfeet, because genetic deletion of AQ4 severely impairs this glymphatic system, disrupts the paravascular flow of Aβ (Iliff et al., 2012), and leads to an increased amyloid burden in the form of both plaques and CAA, and further cognitive impairment in APP/PS1 mice (Xu et al., 2015). In addition, aging has been associated with a decreased perivascular localization of AQ4 and, thus, impaired paravascular clearance of endogenous Aβ in wild-type mice (Kress et al., 2014).

However, these studies have been recently disputed. Using computational models and similar multiphoton microscopy experiments in wild-type and AQ4 null living mice and rats, the Verkman lab has refuted both the hypothesis that arterial pulse-driven convective bulk flow rather than simple diffusion is responsible for the vast part of solute movement through the brain parenchyma, as well as the involvement of AQ4 in solute transport in the rodent brain (Jin et al., 2016; Smith et al., 2017). Another recent study applied computational modeling to 3D-reconstruction of electron microscopy images of the neuropil and concluded than diffusion rather than bulk-flow accounts for the transport of interstitial solutes (Holter et al., 2017). Other computational modeling studies have tried to explain cerebral metabolite clearance by proposing that there is a network of astrocytes connected through AQ4 channels that serve as sites of low resistance to bulk flow, and that the arterial pulsation determines a fast para-arterial transport through dispersion (the combined effect of local mixing and diffusion in the para-arterial space), rather than bulk flow (Asgari et al., 2015, 2016). Studies on AQ in the human AD brain have also yielded conflicting results. Aquaporin 1 (AQ1) immunoreactivity has been shown to increase in close proximity to amyloid plaques and CAA in some studies (Misawa et al., 2008; Hoshi et al., 2012), whereas other authors found no difference in AQ1 immunoreactivity between AD and healthy control subjects, reported AQ1 to be expressed primarily in the white matter rather than the cortical astrocytes, and observed a decrease in expression with aging (Moftakhar et al., 2010). Furthermore, in another study, the researchers only found significantly increased levels of AQ1 by Western blot at early stages of the disease (Braak II; Pérez et al., 2007). With regards to aquaporin 4 (AQ4), no significant differences in protein levels were found between AD and healthy control subjects by Western blot (Pérez et al., 2007), but an enhanced immunoreactivity associated with amyloid plaques and CAA has been reported by other authors (Moftakhar et al., 2010; Hoshi et al., 2012), suggesting the possibility of a redistribution of AQ4 within astrocytes in AD. More recently, in a population-based clinic-pathological study, Zeppenfeld et al. (2017) reported an increased global AQ4 immunoreactivity in AD patients compared to aged and young healthy controls, associated with a loss of perivascular localization that correlated inversely with an increased plaque burden and a higher Braak NFT stage, which seemingly reconciles the human data with the mouse data above.

## Signaling Pathways Involved in Astrocyte Reaction in Alzheimer’s Disease

**Figure 5:**
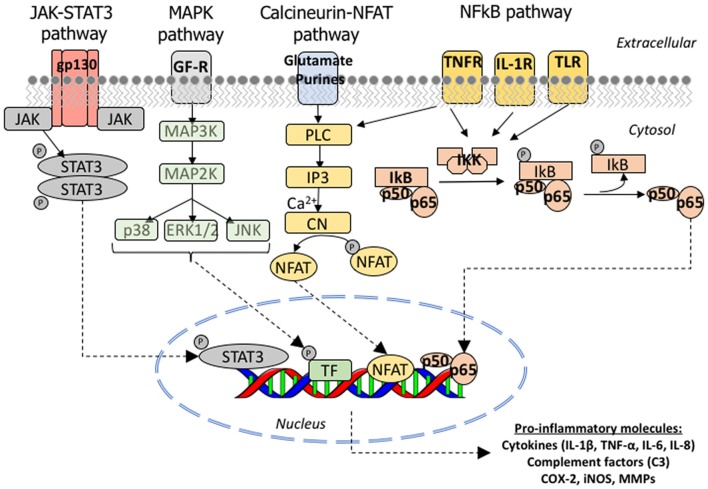
Molecular signaling pathways involved in astrocyte reaction in AD. Cartoon illustrating the JAK/STAT3, MAPK, CN/NFAT and NFκB signaling cascades. *JAK/STAT3 pathway*: the JAKs (JAK 1, 2, 3 and Tyk2) are tyrosine-kinases associated with the intracellular domain of cytokine receptors. The binding of a cytokine ligand to its receptor triggers a dimerization or oligomerization of the associated JAK and this leads to its transphosphorylation and activation. The activated JAK then phosphorylates key tyrosine residues in the cytokine receptor, which leads to the recruitment of STAT (STAT1, 2, 3, 4, 5A, 5B or 6) and a single tyrosine phosphorylation at the Y of its C-terminus. Once phosphorylated, STAT forms homo or heterodimers and is imported to the nucleus, where it complexes with other transcription factors, notably NFkB, to bind its target genes. *MAPK pathway*: the p38 mitogen-activating protein kinase (p38 MAPK) is a family of serine-threonine kinases of which p38α is the most studied due to its ubiquitous expression at high levels. p38α phosphorylates multiple substrates including transcription factors, DNA and RNA binding proteins, other serine-threonine kinases (including GSK3β), and cell cycle and pro-apoptotic proteins. *Calcineurin/NFAT pathway*: the phosphatase function of CN is activated upon the binding of calcium and calmodulin to its regulatory subunit. Once activated, CN removes several phosphate residues from the N-terminus of the transcription factors NFATc (nuclear factor of activated T cells) proteins, named NFATc1 to 4, and exposes their nuclear localization motif, leading to their rapid entry into the nucleus. NFATc targets are largely genes encoding cytokines, growth factors and their receptors, and cell adhesion proteins, as well as many microRNAs. *NFκB pathway*: NFκB is a heterodimeric transcription factor composed of the subunits p65/RelA and p50, which belong to the Rel family of proteins. It is normally located in the cytoplasm in an inactive form due to its binding to the inhibitor IκB, which masks its nuclear localization signal located in the p65 subunit. Activation of IKK complex by certain extracellular signals can dissociate NFκB and IκB by promoting the phosphorylation of the IκB-α, which is a necessary step for IκB-α ubiquitination and subsequent degradation by the proteasome. Once free from IκB, NFκB can translocate to the nucleus and bind to the 10 base pair consensus-sequence GGGACTTTCC GGGRNNYYCC in the promoter of its target genes (NFκB response elements). Abbreviations: CN, calcineurin; COX, cyclo-oxygenase; ERK, extracellular signal-regulated kinases; IL, interleukin; IL1-R, interleukin 1 receptor; iNOS, inducible nitric oxide synthetase; IP3, inositol-3-phosphate; JAK, Janus kinase; MAPK, mitogen-activated protein kinase; MMPs, matrix metalloproteases; NFAT, nuclear factor of activated T cells; PL3, phospholipase 3; STAT, signal transducer and activator of transcription; TLR, toll-like receptor; TNF-α, tumor necrosis factor α; TNF-R, tumor necrosis factor receptor.

To date, *in vivo* and postmortem evidence has implicated four main signaling pathways in the astrocyte reaction in AD: the Janus kinase (JAK)/STAT3, the calcium/CN/NFAT, the NFκB and the MAPK pathways. Figure 5 depicts these molecular cascades that transmit information from extracellular signals to target genes in the nucleus. Other pathways probably involved in astrocyte reaction but with less *in vivo* supporting evidence are calpain and caspase activation.

### JAK/STAT3 Pathway

The JAK-STAT (signal transducer and activator of transcription) is a signaling pathway activated by extracellular cytokines (Mertens and Darnell, 2007). The levels of STAT1 protein were shown to be increased in both the cytosolic and particulated fractions from the cortex of AD patients compared to healthy subjects (Kitamura et al., 1997). Recently, STAT3 immunoreactivity was shown to be increased in the nucleus of GFAP- and vimentin-immunoreactive astrocytes in the APPswe/PS1dE9 and 3xTg mouse models of AD. Lentiviral expression of suppressor of cytokine signaling protein 3 (SOCS3)—a negative regulator of this pathway, which is upregulated by STAT and switches off the activity of JAK—specifically in astrocytes led not only to a decrease in nuclear STAT3 immunoreactivity but also to a reduction of GFAP immunoreactivity, implicating the JAK/STAT3 pathway in astrocytic reaction (Ben Haim et al., 2015).

### Calcium/Calcineurin/NFAT Pathway

The calcium/CN/NFAT pathway links cytosolic calcium levels to gene expression (Crabtree and Schreiber, 2009). CN has been reported to play a central role in AD pathophysiology both in neuronal and astrocytic phenotypes. In neurons, CN excessive activation has deleterious consequences, both functional and morphological. Soluble Aβ oligomers bind to NMDA receptors and increase cytosolic calcium, leading to CN activation and enhanced long term depression (LTD), which contributes to memory impairment. The morphological substrate of CN overactivation is the triad of synaptic loss, dystrophic neurites, and neuron loss (Wu et al., 2010). Inhibiting CN/NFAT pathway in APP/PS1 mice, either by treatment with the CN inhibitor immunosuppressant drug tacrolimus (also called FK-506; Rozkalne et al., 2011) or through the viral-mediated neuronal expression of the specific inhibitor peptide VIVIT, restored this phenotype (Hudry et al., 2012).

Soluble Aβ oligomers also determine a cytosolic calcium overload and CN activation in astrocytes *in vitro*, which leads to an overexpression of GFAP and, subsequently, a reactive phenotype characterized by the release of inflammatory cytokines and the down-regulation of the glutamate transporter GLT-1/EAAT2 (Norris et al., 2005; Abdul et al., 2009). *In vivo* studies also support CN overactivation in astrocytes. Resting calcium level and frequency and synchrony of calcium transients are increased in reactive astrocytes from APP/PS1 mice (Kuchibhotla et al., 2009; Delekate et al., 2014). CN immunoreactivity is increased in hippocampal astrocytes of aged wild-type mice and APP/PS1 mice (Norris et al., 2005). A high activity proteolytic fragment of CN with 45–48 KDa molecular weight range is highly expressed by astrocytes surrounding plaques in human postmortem brain sections (Pleiss et al., 2016). In the human AD hippocampus, CN-Aα and NFAT3 have been shown to increasingly translocate to the astrocyte nucleus as the disease advances, whereas NFAT1 has been shown to translocate to the nucleus mainly in the stage of mild cognitive impairment (Abdul et al., 2009). Inhibition of CN/NFAT pathway in hippocampal astrocytes through the selective viral-mediated expression of VIVIT in astrocytes improved cognitive and synaptic function, reduced glial activation, lowered Aβ levels, increased GLT-1 expression, and reduced glutamate-mediated neuronal excitotoxicity in plaque-bearing AD mice (Furman et al., 2012; Sompol et al., 2017).

### NFκB Pathway

The NFκB pathway is activated by membrane receptor signals from Toll-like receptors, TNFα receptor, T-cell receptor and B-cell receptor. Among NFκB target genes are cytokines (Il-1, IL-6, TNFα, COX-2), complement proteins (i.e., C3), major histocompatibility complex (MHC) class 1 and 2, β-2 microglobulin and APOE (Hayden et al., 2006). Of note, complement fraction C3 has been proposed as a specific marker of a subtype of reactive GFAP-positive astrocytes that are neurotoxic (so-called A1 astrocytes; Liddelow et al., 2017).

Among the stimuli that can trigger this pathway are Aβ_1–40_ and Aβ_1–42_, S100β protein and reactive oxygen species (ROS). *In vitro* experiments in primary culture of rat cortical astrocytes have shown that Aβ at nM or μM concentrations can activate NFκB leading to an upregulation of pro-inflammatory cytokines IL-1 and IL-6 (Bales et al., 1998) and inducible nitric oxide synthetase (iNOS; Akama et al., 1998). The astrocytic secreted S100β protein can also up-regulate iNOS in cultured astrocytes in an autocrine fashion through a NFκB-dependent mechanism (Lam et al., 2001).

Importantly, suppression of NFκB in the APPswe/PS1dE9 ameliorated astrocytic reaction. However, intriguingly, the level of the NFκB inhibitor IκB-α was normal in 3xTg AD mice compared to wild-type littermates, arguing against NFκB activation in this mouse model (Ben Haim et al., 2015). The levels of NFκB p65 protein were shown to be increased in the cortex from AD patients compared to healthy subjects (Kitamura et al., 1997). However, neuropathological studies in AD brains addressing the cell type responsible for this increase have revealed that NFκB immunoreactivity is increased in the cytoplasm and nucleus of neurons, especially those surrounding amyloid plaques or bearing a NFT (Terai et al., 1996; Kaltschmidt et al., 1997; Ferrer et al., 1998). According to these studies the astrocytic NFκB immunoreactivity is either undetectable (Ferrer et al., 1998), only present in the cytoplasm of cortical layer I astrocytes and to a similar extent than in control brains (Terai et al., 1996), or only present in reactive astrocytes around diffuse plaques (Kaltschmidt et al., 1997).

### MAPK Pathway

The mitogen-activating protein kinase (MAPK) pathway is represented by three main families of kinases, all of which are activated by extracellular signals: the p38 MAPK, the c-Jun kinase (JNK), and the extracellular signal-regulated kinases (ERK 1 and 2).

The p38 MAPK together with Jun kinase (JNK) are known as the stress kinases. p38α is activated by very diverse extracellular stimuli including UV light, heat and osmotic shock, oxidative stress, cytokines, chemokines, hormones and growth factors and is thought to have a key role in the cell response to many extracellular threats, leaning the fate of the cell towards survival vs. apoptosis. Inhibition of the p38 MAPK pathway in astrocytes has anti-inflammatory effects in astrocytes *in vitro* (Da Silva et al., 1997; Bhat et al., 2012).

The ERKs are essentially activated by trophic factors and are thought to play an important role in synaptic plasticity. Importantly, a postmortem immunohistochemical study revealed that this pathway is upregulated in the AD brain specifically in GFAP+ reactive astrocytes, but only in the white matter and only at early AD stages corresponding to mild dementia. In moderate and advanced AD dementia, the astrocytic expression was equivalent to non-demented control individuals and predominated in cortical pyramidal neurons, their axons, and plaque-associated dystrophic neurites (Webster et al., 2006). The authors speculated with a protective role of this signaling pathway in astrocytes against early neuronal and synaptic damage.

## Apolipoprotein E Genotype and Astrocyte Reaction

The *APOE*ε4 allele remains the strongest genetic risk factor for the development of AD. Compared to the most common genotype in the general population ε3/ε3, carrying one copy of the ε4 allele increases the risk of developing AD ~2–3 times, whereas homozygous (ε4/ε4) individuals have a ~8–12-fold higher risk. Moreover, the ε4 allele anticipates the onset of AD in a dose-dependent manner (Corder et al., 1993). By contrast, the ε2 allele is protective against the development of AD (Corder et al., 1994; Serrano-Pozo et al., 2015). The main normal function of the APOE is the transport of cholesterol within the brain in the form of high-density lipoprotein (HDL) particles. However, apoE4 has been shown to favor Aβ accumulation by promoting its aggregation in the form of amyloid plaques (Hyman et al., 1995) and soluble Aβ oligomers (Hashimoto et al., 2012), and by reducing Aβ clearance (Castellano et al., 2011). Remarkably, although large clinico-pathological studies have established that the *APOE*ε4 allele does not increase either the burden or the Braak stages of NFTs independently of amyloid plaques (Serrano-Pozo et al., 2015; Farfel et al., 2016), apoE4 has recently been shown to promote tau pathology and neurodegeneration in a mouse model of tauopathy (Shi et al., 2017).

Because, together with microglia, astrocytes are the main source of apoE within the brain, whether the *APOE* genotype has any influence on the astrocyte reaction found in the AD brain is a research topic of growing interest. Using stereology-based quantitative methods in postmortem human AD brain specimens, *APOE*ε4 carriers and non-carriers did not differ significantly in either the number of total, resting (GFAP-negative), or reactive (GFAP-positive) astrocytes (Serrano-Pozo et al., 2013a), the progression of astrocyte reaction along the clinical course of the disease (Serrano-Pozo et al., 2011b), or the proximity of the association between reactive astrocytes and plaques (Serrano-Pozo et al., 2016), but see also (Mathur et al., 2015). However, it is still possible that reactive astrocytes behave differently in different *APOE* genetic backgrounds. For example, *APOE*ε4 KI mice show increased numbers of reactive astrocytes and activated microglia, increased levels of pro-inflammatory cytokines, and decreased levels of synaptic markers after administration of LPS, compared with* APOE*ε3 and *APOE*ε2 KI mice, suggesting that *APOE*ε4 astrocytes may be more susceptible to react to pro-inflammatory stimuli (Zhu et al., 2012). Indeed, E4FAD and P301S/E4 mice (corresponding to the 5xFAD mouse model of brain β-amiloidosis and the P301S tau transgenic mouse of tauopathy under a human *APOE*ε4 KI background, respectively) have a hyperactivated microglia and increased levels of proinflammatory cytokines as compared to the *APOE*ε3 and *APOE*ε2 KI double transgenic mice (Rodriguez et al., 2014; Shi et al., 2017). On the other hand, human inducible pluripotent stem cell (hiPSC)-derived astrocytes from *APOE*ε4/ε4 human subjects exhibit a loss of neurotrophic function with respect to neurons and synapses* in vitro* (Zhao et al., 2017). In another study, *APOE*ε4 astrocytes showed decreased potential to phagocytose synapses, whereas this ability was enhanced in *APOE*ε2 astrocytes compared to *APOE*ε3. However, *APOE*ε4 KI mice had an increased proportion of C1q-tagged synapses (and *APOE*ε2 KI mice a decreased proportion) compared to *APOE*ε3 KI mice, which would make them vulnerable to elimination by microglia (Chung et al., 2016).

## Cross-Talk Between Astrocytes and Microglia

Although astrocytes and microglia share the consideration of glial cells, microglial cells are thought to derive from mesenchymal cells of the yolk sac. Microglia are the innate immune cells of the brain, likewise macrophages in other organs. As such, they are highly dynamic cells that continuously survey the brain tissue in normal conditions, migrate to areas of injury (Nimmerjahn et al., 2005), and phagocytose virus, bacteria and neuronal debris (Fuhrmann et al., 2010). There are multiple lines of evidence supporting the existence of a cross-talk between astrocytes and microglia in AD. First, like astrocytes, activated microglia decorate dense-core amyloid (senile) plaques (Itagaki et al., 1989; Serrano-Pozo et al., 2013b, 2016), where they establish an intimate relationship with astrocytes (Bouvier et al., 2016). Second, there is a strong correlation between the number of activated microglial cells and that of reactive astrocytes, and both parallel disease progression (Serrano-Pozo et al., 2011b). Third, the attenuation of astrocyte reaction around plaques observed in APP/PS1: *GFAP/Vimentin* double knock out mice is associated with increased numbers of activated microglia around plaques (Kraft et al., 2013). Fourth, the “paracrine” secretory function of microglial cells can change astrocyte phenotype and vice versa. Inflammatory cytokines secreted by microglia (i.e., TNFα, IL-1, C1q) can transform neuroprotective resting astrocytes (A2) into neurotoxic (A1). Conversely, astrocytes can release C3 complement fraction via NFκB in response to oligomeric Aβ and C3 can in turn activate microglia through its C3R receptor (Lian et al., 2016). Fifth, microglial processes can be present in excitatory synapses together with astrocyte processes and pre- and post-synaptic neuronal elements, forming a “quadripartite” synapse. Activated microglia can cause direct synaptotoxicity through the secretion of C1q (Hong et al., 2016). Sixth, astrocytes appear to influence the degree of microglial reactivity in mouse models of brain β-amyloidosis (Rodriguez et al., 2014) and tauopathy (Shi et al., 2017) in an apoE isoform-dependent fashion (E4 > E3 > E2). Thus, it is likely that reactive astrocytes and activated microglia do not “play solo”, but act in a concerted fashion.

## Biomarkers of Astrocyte Reaction

Since astrocyte reaction appears to be linked to the neurodegenerative process in AD, it follows that imaging and CSF biomarkers of astrocyte reaction could be useful to improve the accuracy of the clinical diagnosis of AD dementia and monitor and predict the progression of the disease.

With regards to CSF biomarkers, initially a candidate-based approach led researchers to measure the levels of classic astrocyte markers in CSF such as GFAP, S100β, and glutamine synthetase. Compared to healthy controls, GFAP levels have been found to be higher in the CSF of AD, dementia with Lewy bodies (DLB), frontotemporal lobar degeneration (FTLD) and Creutzdfeldt-Jakob disease (CJD) patients, whereas S100β was found to be elevated in CJD, but not in AD. Reports on elevated CSF levels of glutamine synthetase in AD are conflicting (Gunnersen and Haley, 1992; Tumani et al., 1999; Timmer et al., 2015). However, it is a “non-*a priori* hypothesis” proteomic approach that has rendered the most promising CSF biomarker of astrocyte reaction to date: chitinase 3 protein-like 1 (also called YKL-40). This CSF biomarker has been shown to predict progression from normal cognition to MCI and from MCI to AD dementia (Craig-Schapiro et al., 2010). Subsequent studies confirmed a correlation between CSF YKL-40 levels and biomarkers of neurodegeneration, such as CSF total and phospho-tau levels and cortical thickness, at the earliest stages of AD (Antonell et al., 2014; Alcolea et al., 2015). However, elevated CSF YKL-40 levels are not specific of AD; the highest levels appear to occur in sporadic CJD patients, followed by AD and tauopathies (PSP, CBD and Pick’s disease), whereas patients with vascular dementia and with Parkinson disease dementia or DLB have been reported to have normal levels (Llorens et al., 2017). Importantly, YKL-40 is expressed by GFAP-immunoreactive astrocytes near plaques and CAA-laden vessels (Craig-Schapiro et al., 2010; Llorens et al., 2017), but also in white matter fibrous astrocytes and in random cortical protoplasmic and perivascular astrocytes (Llorens et al., 2017). A recent postmortem quantitative neuropathological study in AD and other tauopathies has described that YKL-40 is only expressed by a subset of GFAP-immunoreactive astrocytes and that there is a positive correlation between YKL-40 and tau immunoreactivities, in agreement with the correlations found in CSF (Querol-Vilaseca et al., 2017).

The development of PET radiotracers specific for astrocyte reaction has proven to be more challenging than for activated microglia. While PET radioligands of the translocator protein 18 kDa (TSPO), also called peripheral benzodiazepine receptor (PBR), have been used to depict microglial activation *in vivo* for almost two decades (Cagnin et al., 2001), no such established radiotracer exists for reactive astrocytes. However, it should be noted that TSPO is not only up-regulated in activated microglial cells but also in reactive astrocytes, and that it is also expressed by endothelial and vascular smooth muscle cells (Cosenza-Nashat et al., 2009). Recently, [^11^C]-deuterium-L-deprenyl ([^11^C]-DED), a modified inhibitor of the monoamino oxidase B (MAO-B) enzyme, has been proposed as a PET imaging biomarker of astrocyte reaction (Carter et al., 2012; Schöll et al., 2015; Rodriguez-Vieitez et al., 2016). Indeed, MAO-B was shown to be up-regulated in GFAP-immunoreactive astrocytes many years ago (Nakamura et al., 1990; Jossan et al., 1991). However, the specificity of this radiotracer requires further validation because MAO-B is also expressed by neurons and the contribution of each cell type to the radiotracer uptake remains to be clarified.

The results of astrocyte-specific transcriptomic studies in the human AD brain and AD mouse models will inform the development of new CSF and PET imaging biomarkers specific of astrocyte reaction (Zamanian et al., 2012; Orre et al., 2014; Liddelow et al., 2017).

## Conclusion

In summary, recent evidence directly implicates astrocytes in the pathophysiology of AD and supports the idea that astrocyte reaction against amyloid plaques and NFTs leads to a loss of their neurotrophic potential and a gain of neurotoxic properties. Large gaps of knowledge remain regarding the molecular pathways involved in this reaction and its functional consequences for both astrocytes themselves, and neurons and their synapses. Future research should also address the implication of each AD pathological hallmark in astrocyte reaction, and further characterize the cross-talk between astrocytes and microglia and the influence of the *APOE* genotype. Technological advances will help answer some of these questions, including some new *in vitro* tools (human induced pluripotent stem cells (hiPSCs)-derived astrocytes, 3D-cultures, brain organoids, protocols of isolation and purification of astrocytes from the adult mouse and human brains), recently developed unbiased and high-throughput molecular biology techniques (i.e., single-cell RNAseq), new mouse models and gene delivery approaches for astrocyte-specific manipulations, and more specific CSF biomarkers and PET radiotracers for *in vivo* studies of astrocyte reaction in the human brain.

## Author Contributions

AS-P and BGP-N designed this review outline, performed literature review, prepared the figures and wrote the manuscript.

## Conflict of Interest Statement

The authors declare that the research was conducted in the absence of any commercial or financial relationships that could be construed as a potential conflict of interest.
